# Immunological signatures from irradiated cancer-associated fibroblasts

**DOI:** 10.3389/fimmu.2024.1433237

**Published:** 2024-09-06

**Authors:** Rodrigo Berzaghi, Kristian Gundersen, Brede Dille Pedersen, Amalie Utne, Nannan Yang, Turid Hellevik, Inigo Martinez-Zubiaurre

**Affiliations:** ^1^ Department of Clinical Medicine, Faculty of Health Sciences, UiT The Arctic University of Norway, Tromsø, Norway; ^2^ Department of Radiation Oncology, University Hospital of North Norway, Tromsø, Norway; ^3^ Department of Community Medicine, Faculty of Health Sciences, UiT The Arctic University of Norway, Tromsø, Norway

**Keywords:** cancer-associated fibroblasts, CAFs, immunosuppression, ionizing radiation, radiotherapy, non-small cell lung cancer, NSCLC, tumor microenvironment

## Abstract

**Introduction:**

Cancer-associated fibroblasts (CAFs) are abundant and influential elements of the tumor microenvironment (TME), giving support to tumor development in multiple ways. Among other mechanisms, CAFs are important regulators of immunological processes occurring in tumors. However, CAF-mediated tumor immunomodulation in the context of radiotherapy remains poorly understood. In this study, we explore effects of radiation on CAF-derived immunoregulatory signals to the TME.

**Methods:**

Primary CAF cultures were established from freshly collected human NSCLC lung tumors. CAFs were exposed to single-high or fractionated radiation regimens (1x18Gy or 3x6Gy), and the expression of different immunoregulatory cell-associated and secreted signaling molecules was analyzed 48h and 6 days after initiation of treatment. Analyses included quantitative measurements of released damage-associated molecular patterns (DAMPs), interferon (IFN) type I responses, expression of immune regulatory receptors, and secretion of soluble cytokines, chemokines, and growth factors. CAFs are able to survive ablative radiation regimens, however they enter into a stage of premature cell senescence.

**Results:**

Our data show that CAFs avoid apoptosis and do not contribute by release of DAMPs or IFN-I secretion to radiation-mediated tumor immunoregulation. Furthermore, the secretion of relevant immunoregulatory cytokines and growth factors including TGF-β, IL-6, IL-10, TNFα, IL-1β, VEGF, CXCL12, and CXCL10 remain comparable between non-irradiated and radiation-induced senescent CAFs. Importantly, radiation exposure modifies the cell surface expression of some key immunoregulatory receptors, including upregulation of CD73 and CD276.

**Discussion:**

Our data suggest that CAFs do not participate in the release of danger signals or IFN-I secretion following radiotherapy. The immune phenotype of CAFs and radiation-induced senescent CAFs is similar, however, the observed elevation of some cell surface immunological receptors on irradiated CAFs could contribute to the establishment of an enhanced immunosuppressive TME after radiotherapy.

## Introduction

External beam radiotherapy (RT) represents one of the most effective and most used treatment modalities in oncology (1, 2). During recent years, RT has attracted considerable interest as a potential combination partner to immunotherapy owing to its widespread clinical availability, its predictable safety profile, and its potential to serve as anti-tumor immunostimulant (3, 4). The immunoregulatory power of RT has been widely explored by many groups in both preclinical and clinical models. Among other effects, tumor irradiation promotes upregulation of MHC molecules on tumor cells, with the accompanying presentation of novel antigenic determinants to the immune system (5, 6). Furthermore, RT-induced killing of tumor cells may provoke release of tumor-associated antigens to the circulation, thus directly contributing to enhancement of anti-tumor antigenicity (7). Besides, RT has the power to trigger a particular program of cell demise known as immunogenic cell death (ICD), which contributes to anti-tumor immune adjuvanticity (8). ICD is associated with the emission of several damage-associated molecular patterns (DAMPs), including extracellular ATP and high motility group protein1 (HMGB-1); translocation of the endoplasmic reticulum chaperone calreticulin (CALR) to the plasma membrane; and active secretion of numerous immunostimulatory or chemotactic cytokines including type I interferons (IFN-I) and C-X-C motif chemokine ligand 10 (CXCL10) to the tumor microenvironment (9–11). In line with these observations, RT has been shown to synergize with immune checkpoint blockers (ICBs) in different immunocompetent tumor mouse models (12, 13).

Despite such plenitude of preclinical studies providing solid foundations for the use of RT as a combinatory partner for immunotherapy, numerous clinical studies have failed to reveal a therapeutic benefit over either treatment modality alone (14, 15). Such observations in the clinics reveal the existence of unidentified obstacles to the successful implementation of RT/IT combination regimens. Additional knowledge on both immunosuppressive and immunostimulatory effects induced by RT on different tumor elements is needed to ascertain the true potential of RT to function as an effective *in situ* cancer vaccine (16, 17).

Cancer-associated fibroblasts (CAFs) are one of the most abundant elements of the tumor microenvironment and have been shown to be directly implicated in malignant progression in different ways (18, 19). Besides sending signals that promote tumor cell growth and therapy resistance, CAFs also participate in the development of desmoplastic reactions in tumors and tumor immune evasion (20). In fact, one of the best-characterized traits of CAFs is their prominent role as tumor immuno-suppressors of the TME (21). CAF-mediated immunoregulation is primarily mediated via the release of soluble cytokines and growth factors, however, other known mechanisms used by CAFs to regulate tumor immunity comprise exosome release, direct cell-cell mediated interactions, and regulation of extracellular matrix and tissue stiffness (22).

In the context of radiotherapy, the eventual role played by CAFs on therapeutic outcomes remains controversial (23). While some studies claim that RT has negative effects on CAFs by inducing impaired motility and growth arrest and by abrogating some of their pro-tumorigenic effects (24–26), others argue that radiation exposure to fibroblasts promotes their conversion into a more activated and aggressive phenotype (27). Of note, recent *in vitro* studies have shown that CAF-mediated immunosuppressive functions exerted over different immune cell types such as T cells (28), dendritic cells (29), NK cells (30) or macrophages (31) seem to be maintained after radiation (32). Nevertheless, the field is still in need of further knowledge that can help to better understand CAF responses to ionizing radiation and to further elucidate the potential role that CAFs may play in tumor radio-resistance. In this work, we explore potential participation of CAFs in anti-tumor adjuvanticity in the radiotherapy setting. CAF-derived immunological signatures have been investigated in the form of ICD-like induction, generation of DAMPs, secretion of soluble immunoregulatory factors, intracellular regulation of NF-κβ, and STAT-1 signaling, including surface expression of immunoregulatory receptors/ligands. Radiation-induced changes in checkpoint ligands have been demonstrated also in *in vivo* models.

## Materials and methods

### Human material, CAF isolation, and cell cultures

Human lung CAFs were isolated from resected non-small cell lung cancer (NSCLC) tissues from patients undergoing surgery at the University Hospital of Northern Norway (UNN), as previously described (24). For this study, lung tumor samples from five randomly selected patients (with patient characteristics described in **Supplementary Table 1**) were collected under written informed consent and performed according to ethical guidelines and regulations under the approval of the Regional Ethical Committee of Northern Norway (REK Nord 2016/2307). Briefly, CAFs were isolated by enzymatic digestion of tissues and the outgrowth method, and phenotypically characterized by the expression of Alpha Smooth Muscle Actin (α-SMA) and fibroblast activation protein-α (FAP), as described previously (24). Isolated CAFs were cultured in DMEM high glucose basal medium (Sigma-Aldrich, St Louis, MO, USA, Cat. # D5796) supplemented with 10% FBS, 100 U/mL penicillin/100 μg/mL streptomycin. Cells were used for experimentation at low passage numbers (3-6). Human lung adenocarcinoma cancer cell lines A549 (CCL-185) were purchased from LGC Standards AB (Borås, Sweden), and cultured in RPMI basal medium (Sigma-Aldrich, St Louis, MO, USA, Cat. # D5796) supplemented with 10% FBS, 100 U/mL penicillin/100 μg/mL streptomycin.

### Irradiation of cell cultures

Adherent cells were kept in T-75 cell culture flasks and plated in 6-well plates for a minimum of one day before irradiation. Cells were irradiated with high-energy (MV) photons using a clinical Varian linear accelerator, as previously described (24). Two different radiation regimens were used, single-high dose of 18 Gy, or a fractionated regimen of (3x6 Gy) delivered at 24-hour intervals. Standard parameters for dose delivery were beam quality of 15 MV, depth 30 mm, dose rate of 6 Gy/min, field sizes of 20x20 cm, and gantry position at 180°.

### Western blots

Whole-cell extracts were prepared in RIPA buffer (Cell Signaling, Boston, MA, USA) plus Complete Protease and Phosphatase Inhibitor Cocktail (ThermoFisher, Cat. # 78440). Total cell-associated proteins were separated by 10% SDS-polyacrylamide gel electrophoresis (PAGE) and transferred onto a PVDF membrane. Next, the membrane was blocked with 1% BSA in Tris-buffered saline, 0.1% Tween 20 (TBS-T) for 2h at 20°C, and then incubated (overnight, 4°C) with primary antibodies (**Supplementary Table 2**) diluted 1:1000 (in TBS-T with 1% BSA). Subsequently, the membrane was washed (5x) in TBS-T and then incubated with an anti-rabbit or anti-mouse HRP-conjugated secondary antibody (diluted 1:2000; Cell Signaling; #7074 and #7076, respectively) for 1h at 20°C. Finally, proteins transferred to the membrane were visualized with Enhanced Chemiluminescence at ImageQuant LAS4000 CCD (GE Healthcare Bio-Sciences, PA, USA). Relative intensity was assessed using ImageJ software.

### β-galactosidase and apoptosis assay

CAFs were plated in 6-well plates (10,000 cells/well) the day before photon irradiation. Seven days after radiation treatment, cultures were fixed with formaldehyde 4% for 10-15 min at 20°C. Next, β-galactosidase (5-bromo-4chloro-3-indolyl- B-D-galactopyranoside) staining was performed using “Cellular Senescence Assay” (Sigma Aldrich, St. Louise, MO, USA; Cat. # KA0002), following instructions from the manufacturer. Randomly selected fields from the wells were assessed under a light microscope and senescent cells (stained in blue) were counted. In parallel with the β-galactosidase staining procedure, matched cells were assessed for activation of caspases 3 and 7 using “CellEvent™ Caspase-3/7 Green Flow Cytometry Assay Kit” (ThermoFisher Scientific, Waltham, MA, USA; Cat. # C10740) and analyzed by flow cytometry.

### Quantitative cytokine release by ELISA

Quantitative determinations of IFN-β and CXCL10 in CAF-conditioned medium (CAF-CM) were determined using ELISA kits (R&D Systems, Minneapolis, MN, USA), according to the manufacturer’s instructions. Briefly, CAFs (at low passage numbers) were cultured in T-75 tissue culture flasks in DMEM (with 10% FBS) and exposed to one or three medium-high radiation doses (1x6 Gy; 3x6 Gy) or one single-high-dose of IR (1×18 Gy). CAF-incubation media was conditioned (at 37°C) during the first 48h after radiation exposure and between days 4 and 6 after IR treatment, then collected, spun down by centrifugation (2000x g, 4°C, 10 min), filtrated (Ø = 0.45 µm) and stored at -80 °C until used. CAF-CM samples (diluted 1:2) were analyzed for the content of IFN-β and CXCL10, using human IFN-β and CXCL10 ELISA Kits (R&D systems, Cat#: DY814-05 and DIP100, respectively). Protein absorbance at 450 nm for each sample was analyzed by SpectraMax Plus 384 Microplate Reader (Molecular Devices, CA, USA).

### Multiplex protein arrays

A panel of eight specific proteins, including cytokines and chemokines, was measured in the irradiated (1×18 Gy and 3×6 Gy) or non-irradiated CAF-CM from five different and randomly selected donors, by immune-based protein arrays. A customized human cytokine Multiplex kit (Cat. no. LXSHM-09; R&D Systems, Minneapolis, MN, USA) was used to define protein concentrations of IL-4, IL-6, IL-10; CCL2, CXCL12, CXCL8, VEGF-A, and TGF-β. All samples were analyzed in duplicates and 1:2 or 1:4 dilutions. Quantitative protein measurements were performed by using the Luminex Bio-Plex 200 system (Bio-Rad, Hercules, CA, USA). Measured protein concentrations were normalized with cell numbers at specific culture conditions and expressed as pg/mL/10^6^ cells.

### Quantitative cell surface marker expression by flow cytometry

CAF surface markers were analyzed by flow cytometry on BD FACSAria III using the FlowJo software, Ver.7.2.4 (Tree Star, Ashland, OR, USA). Briefly, CAF cultures (3×10^5^ cells/condition) were labeled with panels of specific antibodies (**Supplementary Table 2**) for each marker phenotype (Miltenyi Biotec). Isotype controls consisted of REA control and IgG2a (Cat. no. 130-113-450 and 130-104-612, respectively). Data were obtained by flow cytometry using the following gating strategy: a) cells gated according to their scatter properties (FSC-A vs SSC-A), b) doublets exclusion (SSC-H vs SSC-W), and c) analyzed by percentage of total live cells expressing calreticulin, CD276, CD273, CD73, HLA-DR, PD-L1, OX40L, CD178, and CD253 surface markers.

### *In vivo* tumor models

Female C57BL/6J mice (age 6-8 weeks), weighing 23.3 ± 2.0 g (upon arrival) were purchased from Charles River (Sulzfeld, Germany), and acclimatized in the local animal facility for a minimum of five days before experimentation. All procedures and experiments involving animals were conducted strictly according to regulations by the Federation of European Laboratory Animal Science Association (FELASA) and were approved by the National Animal Research Authority (permission ID 6373, 6942, and 7873). The LLC-LU2 mouse lung carcinoma cells were obtained from the American Type Culture Collection (Rockville, MD, USA) and cultured in DMEM high glucose basal medium (Sigma-Aldrich, St Louis, MO, USA, Cat. # D5796) supplemented with 10% FBS, 100 U/mL penicillin/100 μg/mL Streptomycin plus Blasticidin (10ng/mL). All cancer cells used for tumor implantation were tested for pathogens by Idexx Bioanalytics (Mice Comprehensive test). For transplantation purposes, LLC-LU2 cells were prepared in RPMI culture medium plus Matrigel (1:1, GelTrex, Thermo Fisher Scientific, Cat. #A1413202) and injected (5x10^5^/mouse) subcutaneously into the right flank of animals. Tumors were measured (3X/week) using a digital caliper, and tumor volumes were calculated by the modified ellipsoidal formula (V= ½ (Length x width^2^). Animals were sacrificed on the day of irradiation (baseline) or 7 days after irradiation.

### Treatment planning for small animal radiotherapy

Sedated animals with subcutaneous tumors established on the right flank were placed in the feet-first-decubitus-left (FFDL) position on the treatment couch; connected to the anesthesia system and aligned according to the lasers in the cabinet. Upon acquiring a cone beam CT-scan of the tumor region, resulting DICOM-images were exported into the “Small Animal Radiotherapy Advanced Treatment Planning” (SmART-ATP) software (SmART Scientific Solutions B.V) (33), and structure densities were assigned, as a prerequisite for meaningful dose calculations. Next, tumors and organs-at-risk (OAR) were contoured; isocenter, beam-angles, radiation-dose, and collimator-size defined, and finally, 3D dose-calculations and plan-evaluations in dose-volume-histograms were activated. The accepted treatment plan was exported back to the Pilot XRAD PC for automatic execution of the radiation plan.

### Precision image-guided radiotherapy to murine tumors

Murine tumors were exposed to CT-imaging and focused-beam ionizing radiation by means of the image-guided photon-irradiator X-RAD 225Cx platform (Precision X-Ray irradiation (PXI), Madison, USA), which includes on-board imaging by cone beam CT and an image-guided treatment planning system (SmART) (PXI). Radiation treatments started when inoculated tumors reached diameters of 5-6 mm (~100 mm^3^; ~8-10 days post-implantation). As preparation for tumor irradiation, i.e. before being positioned on the treatment couch, mice received continuous isoflurane gas anesthesia via induction in an anesthesia chamber (0.5 L/min oxygen with 4% isoflurane). During irradiation, i.e. while on the treatment couch, mice received continuous isoflurane anesthesia gas via a nose cone (0.4 L/min oxygen with 2% isoflurane). Based on a preplan-protocol developed in the SmART-ATP treatment planning system and automatic treatment execution on the Pilot PC, a single dose of 12 Gy was delivered precisely to each tumor, by means of two circular opposing photon beams with maximum energy 225 kV, beam collimator-size Ø=10 mm and dose-rate 3 Gy/min. The preplanned protocol was developed to specifically irradiate tumors on the right flank, and secure efficient delivery of high-precision radiation to murine tumors in large experiments, consisting of several animals (n=10) per experimental group, and several treatment groups (n=3), of which animals had been randomly selected. The preplan strategy requires tumors of similar size and location, accurate (image-guided) positioning for each animal, and isocenter focused on the planning target volume (PTV) in each animal/tumor. Structural imaging measurements (MRI and/or CT), as well as digital caliper measurements, were used for monitoring tumor growth post-RT until experimental endpoint.

### Histology and immunohistochemistry

Excised tumors were directly immersed into ice-cold 4% paraformaldehyde (PFA) in PBS and stored (24–48) h at 4 °C in 2% PFA/PBS before paraffin-embedding. Fixed tumor tissues were thereafter cut into thin sections (5 μm) with a cryostat, and sections were deparaffinized and rehydrated before antibody labeling. Immunohistochemistry assays for CD73 and CD276 were performed on the Ventana Discovery-Ultra automated immuno-stainer (Ventana Medical Systems, Tucson, AZ). Deparaffinization and on-board antigen retrieval were performed for 24 min at 100°C with CC1 reagent, an EDTA-based proprietary Ventana solution (pH 8.0–8.5). CD73 and CD276 anti-rabbit polyclonal antibodies (anti-mouse B7-H3 rabbit polyclonal antibody, Thermo Fisher Scientific, Cat#.: PA5-141121; and anti-CD73 rabbit polyclonal, Nordic Biosite, Cat#: GTX113509, dilution 1/50) were applied and incubated for 32 minutes. Stained slides were developed using Ultramap anti-rabbit HRP (Cat#760–4315, Ventana) and detected using ChromoMap DAB (Cat#760–159, Ventana).

### Statistical analysis

All statistical analyses were performed using GraphPad Prism (GraphPad Software, Inc., La Jolla, CA). Comparison of data between experimental groups was analyzed using the Brown-Forsythe and Welch ANOVA test, and significance values were adjusted by Dunnett’s T3 correction for multiple comparisons. Outcomes of Western blot experiments were analyzed using the Two-way ANOVA test, and significance values were adjusted by Dunnett correction for multiple comparisons. The level of significance was set at *p* < 0.05. Results were presented in graphs, where each donor was plotted as an individual dot in the dataset.

## Results

### Effects of radiation on CAF viability

**Figure 1:**
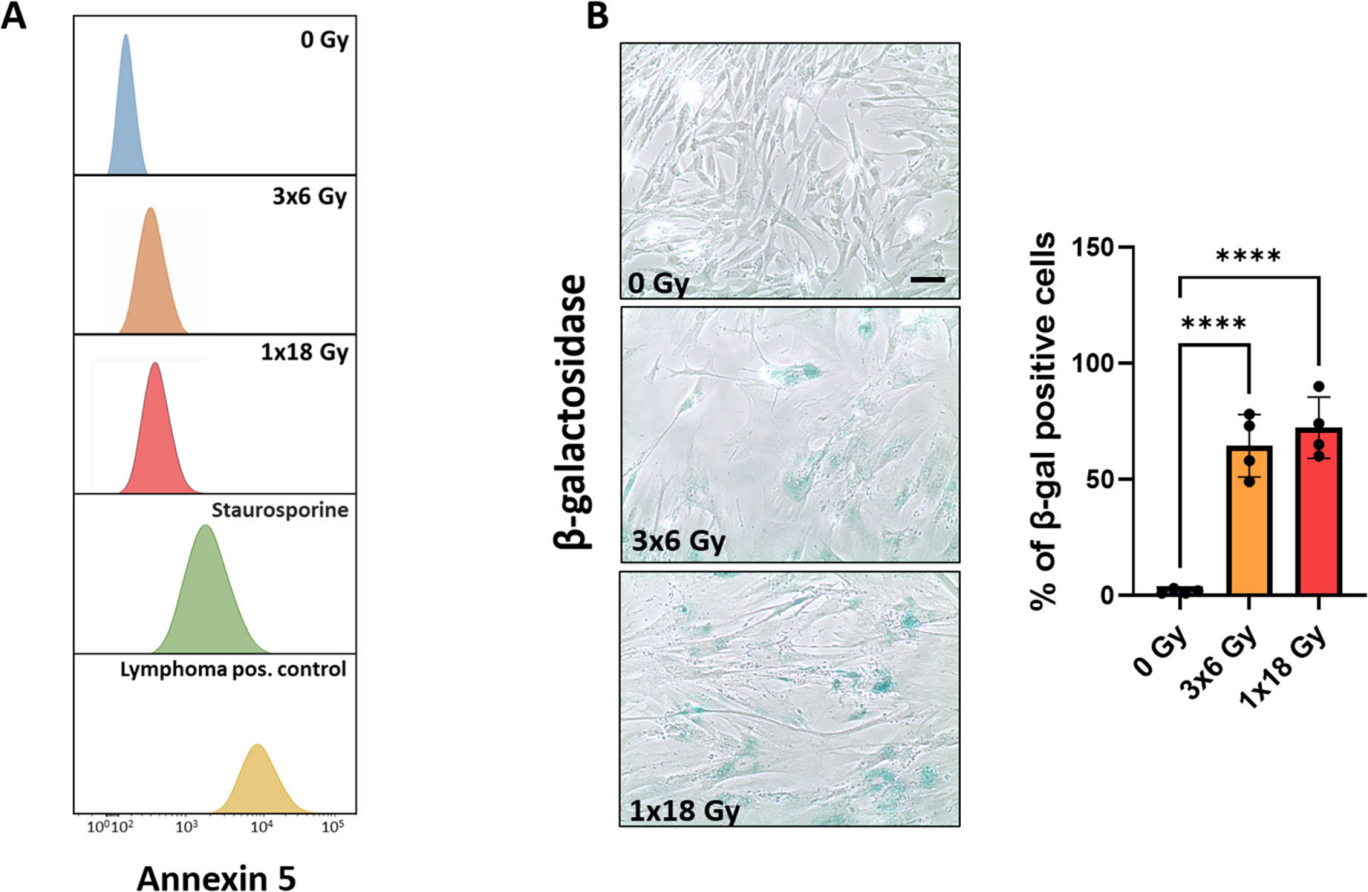
Effects of IR on CAF viability. Induction of cell senescence and apoptosis. Primary Human lung CAF cultures were checked for apoptosis (TUNEL assay—panel **A**) and senescence (β-Galactosidase assay – panel **B**) on day five after first radiation exposure. **(A)** Cell survival and apoptosis were measured by the TUNEL assay and assessed flow cytometry. Positive controls included human lymphoma cells treated with Camptothecin or CAFs treated with the pro-apoptotic agent Staurosporine. **(B)** Premature cell senescence (blue-stained cells) was induced by fractionated-medium and high-dose radiation regimens. Scale Bar = 15 μm. ****Statistically significant, with p<0.001.

Initial experiments were directed to explore the effects of radiation exposure on CAF viability. Radiation regimens comprised fractionated medium-high radiation doses (3x6Gy) and single-high dose radiation (1x18Gy). The chosen radiation doses in this study are relevant in the clinical context, as such doses are frequently used in hypofractionated protocols related to stereotactic ablative body radiotherapy (SBRT) regimens for lung cancer (34–37). Our data clearly indicate that CAFs do not succumb to such high-dose radiation exposure, as demonstrated by negligible induction of apoptosis (**Figure 1A**). Instead, both radiation regimens are able to induce cellular senescence, as measured by intracellular β-galactosidase expression (**Figure 1B**). Senescent CAFs display a strikingly different phenotype than proliferating CAFs, with more elongated and flattened cell morphology (**Figure 1B**). Of note, no cell detachment was observed during the first seven days post-IR treatment.

### Ionizing radiation does not trigger the release of DAMPs from CAFs

**Figure 2:**
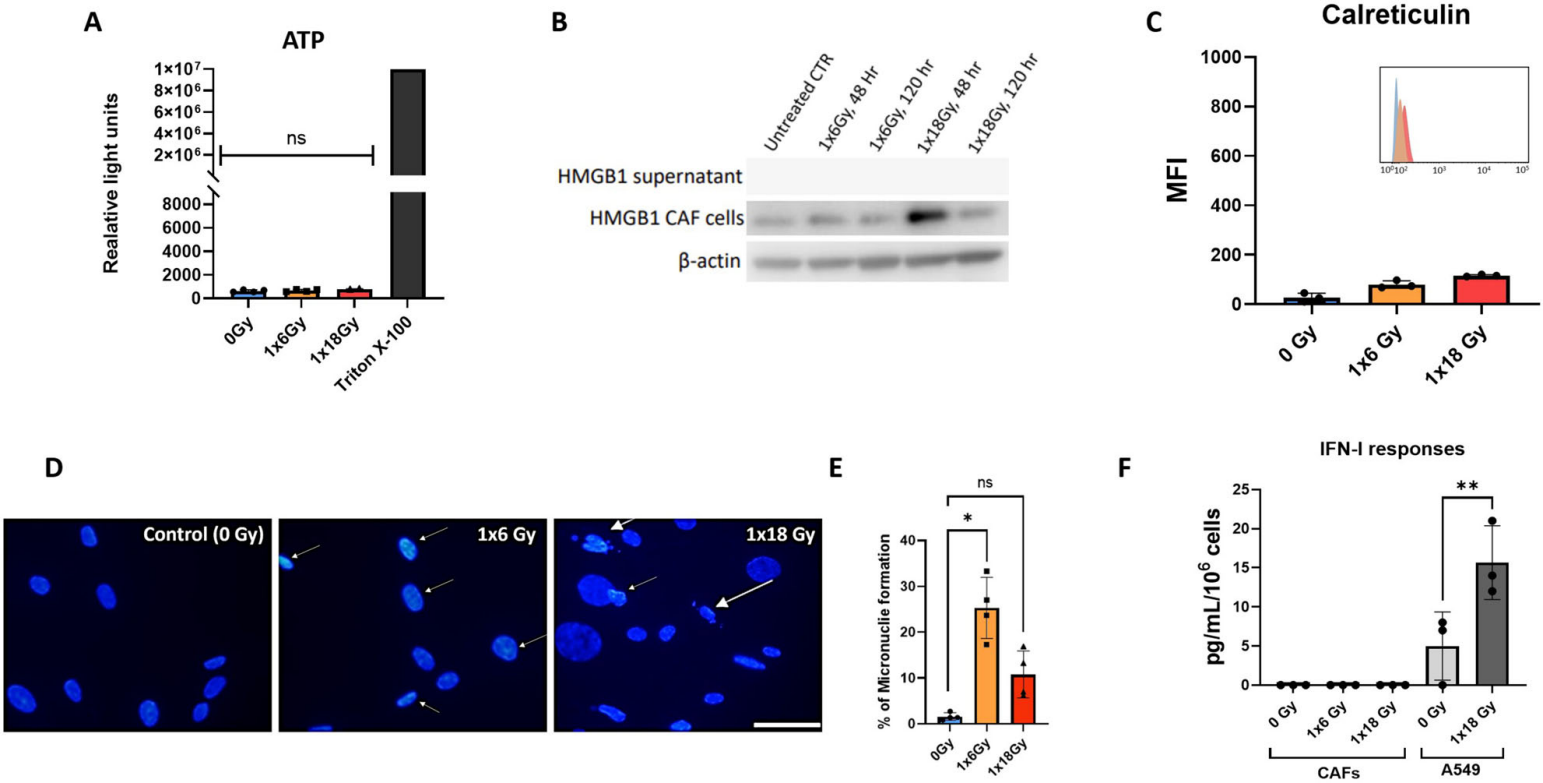
CAF irradiation and generation of DAMPs. **(A)** Levels of extracellular ATP in irradiated Human lung CAF cultures were measured 24h post-radiation treatment. Cells treated with triton X-100 for 1h were used as positive control. Bars represent mean value (± SD) from two different CAF donors; **(B)** Expression of HMGB1 in Human lung CAF cell lysates and supernatants was analyzed by Western blots at 48h and 5 days post-radiation treatment; **(C)** Cell surface calreticulin expression on irradiated Human lung CAFs measured by flow cytometry. Bar graphs represent mean (± SD) values from flow cytometry analysis of 3 CAF donors, measured independently. **(D)** Micronuclei formation in irradiated Human lung CAFs measured by DAPI nuclear staining. **(E)** Bar graphs represent percentage of micronuclei positive cells (± SD) analyzed from 4 CAF donors, measured independently. **(F)** Effects of radiation on secretion of IFN-β by Human lung CAFs and A549 cells were analyzed by ELISA assay. IFN-β secretion in supernatants was determined 48h post-irradiation. Welch ANOVA test and p-values were determined between control and irradiated CAFs. *: Statistically significant, with p<0.05. **: Statistically significant, with p<0.01. ns: Not significant.

Given the relevance of immunogenic cell death (ICD) in the context of radiation-induced anti-tumor immunogenicity (38), we next explored the potential release of DAMPs from irradiated CAFs. Analysis of this issue comprises a) autophagy-dependent secretion of ATP (**Figure 2A**); b) determination of extracellular release of nuclear high mobility box group-1 (HMGB-1) (**Figure 2B**); and c) translocation of the endoplasmic reticulum chaperone calreticulin to the cell surface (**Figure 2C**). Collectively, our data demonstrate that ICD-like responses are not induced in CAFs following exposure to ionizing radiation. Exposure of CAF cultures to hydrogen peroxide (100μM) or Staurosporine (1μM) was used as positive controls, however, although these treatments were able to induce apoptosis in CAFs, none of such treatments were able to induce detectable levels of cell surface calreticulin or extracellular HMGB-1.

The presence of cytosolic DNA is known to initiate pro-immunogenic interferon type I responses via activation of cGAS and STING pathways (39). To shed light on potential IFN responses in CAFs, cells were analyzed for micronuclei formation by DAPI staining, six days after radiation exposure. Both radiation regimens, i.e. (1x6Gy) and (1x18Gy), were able to induce micronuclei formation in a subset of CAFs, accounting for 24% and 9% positive cells, respectively (**Figures 2D, E**). Under such circumstances, we decided to investigate potential secretion of IFN-β into the culture medium from irradiated CAFs. However, in line with previous results on CAF-related DAMPS, we were unable to detect IFN-β in cell supernatants of neither untreated nor irradiated CAFs, even after concentrating samples 20x by ultrafiltration (**Figure 2F**), as shown earlier by our group (29).

### Radiation effects on STAT-1 and NF-κB signaling

**Figure 3:**
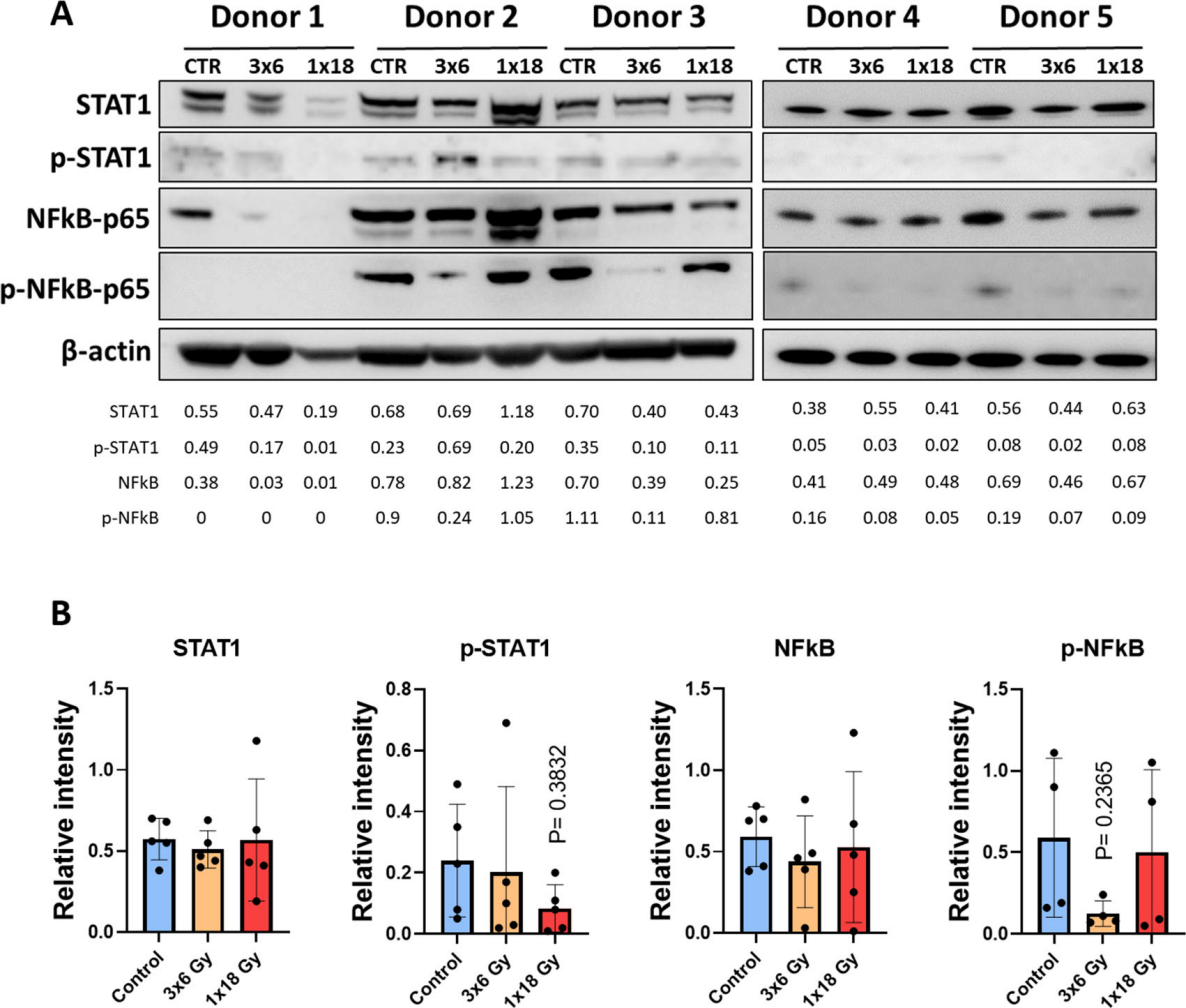
Radiation-induced effects on STAT-1 and NF-κB pathways in CAFs. **(A)** Western blot analysis, using anti-STAT1, p-STAT1 (T701), NF-κB/p65, and p-NF-κB/p65 (S536) on irradiated and non-irradiated Human lung CAF whole cell lysates were analyzed 5 days after irradiation. Results were normalized against β-actin expression. **(B)** Relative intensity of the bands corresponding to panel, determined by densitometry, is shown as a bar graph. Data represent mean (± SD) values from 5 different CAF donors. Two-way ANOVA test and p-values were determined individually between non-irradiated CAFs and the two irradiated CAF groups.

Signal transducer and activator of transcription 1 (STAT-1) and nuclear factor kappa-light-chain-enhancer of activated B cells (NF-κB) are transcription factors involved in the regulation of cytokine production, cell survival, and inflammatory responses (40, 41). NF-κB is involved in cellular responses to stimuli such as stress, cytokines, free radicals, heavy metals, or irradiation, and participates importantly in the regulation of immune (and inflammatory) responses to infection and tissue damage (42). STAT-1 is particularly important in the cellular response to inflammation. It’s activation normally leads to increased expression of interferon-stimulated genes (43) and has been suggested as a crucial factor for cell sensitivity to ionizing radiation (44). Given the relevance of such pathways on potential cytokine responses following radiation-induced cell stress, we studied STAT-1 and NF-κB phosphorylation on CAFs exposed to radiation. As shown in **Figure 3**, that neither STAT-1 nor NF-κB are substantially regulated after radiation exposure in CAFs, 5 days after irradiation. Interestingly, the data shows a near-significant down-regulation of NF-κB phosphorylation in the experimental group receiving fractionated medium-doses of IR (3x6Gy). This outcome may translate into reduced expression of certain signals by CAFs treated with this specific radiation regimen.

### Effects of radiation on secretion of CAF-derived immunomodulatory cytokines and chemokines

**Figure 4:**
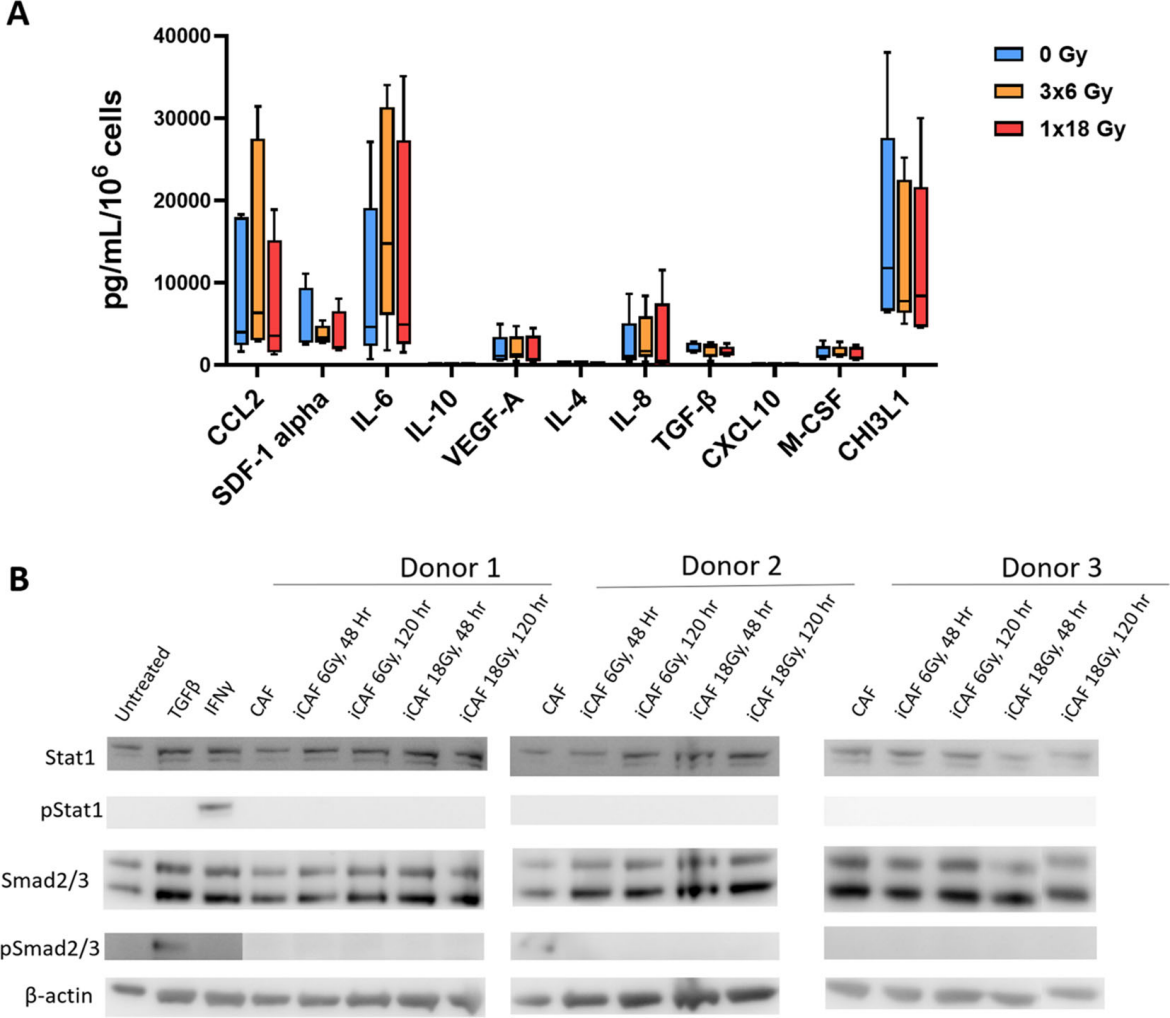
Secretion of inflammatory and immunoregulatory factors by irradiated Human lung CAFs. **(A)** The release of eleven subjectively selected inflammatory/immunoregulatory factors from CAFs was measured in CAF-CMs by ELISA and multiplex protein arrays, 5 days after radiation treatment. Mean values from five different CAF donor samples are shown. **(B)** Levels of active IFNs and TGF-β in CAF-CM were examined by checking induction of STAT-1 and Smad2/3 phosphorylation in A549 cells upon exposure to CAF-CM. Cultured A549 cells were exposed for 3h to CAF-CM, before cell lysis. Figure shows outcomes from 3 unrelated CAF donors.

CAFs are known to mediate most of their immunoregulatory functions in a paracrine way via the secretion of soluble signaling molecules into the TME, including proinflammatory cytokines, chemokines, and growth factors. A panel of eleven well-studied CAF-derived immunoregulatory factors (both immunostimulatory and immunosuppressive) were analyzed in the conditioned medium of CAF cultures before and after irradiation. As shown in **Figure 4A**, secretion of all studied inflammatory mediators remains nearly unchanged in CAFs exposed to radiation. Of note, the levels of some cytokines, such as IL-10 and IL-1β, were under the detection limit of the assay for both untreated and irradiated CAFs. Furthermore, conditioned medium from irradiated and untreated CAFs was applied to A549 lung adenocarcinoma tumor cells to explore activation of STAT-1 and Smad2/3-dependent pathways via secretion of IFNs and TGF-β from CAFs respectively. Exposure of A549 cells to recombinant IFN-γ and TGF-β1 was able to induce phosphorylation of STAT-1 and Smad2/3 respectively. However, none of the CAF-CMs was able to induce measurable activation levels of the mentioned transcription factors (**Figure 4B**).

### Effects of radiation on surface expression of immunoregulatory receptors and ligands

**Figure 5:**
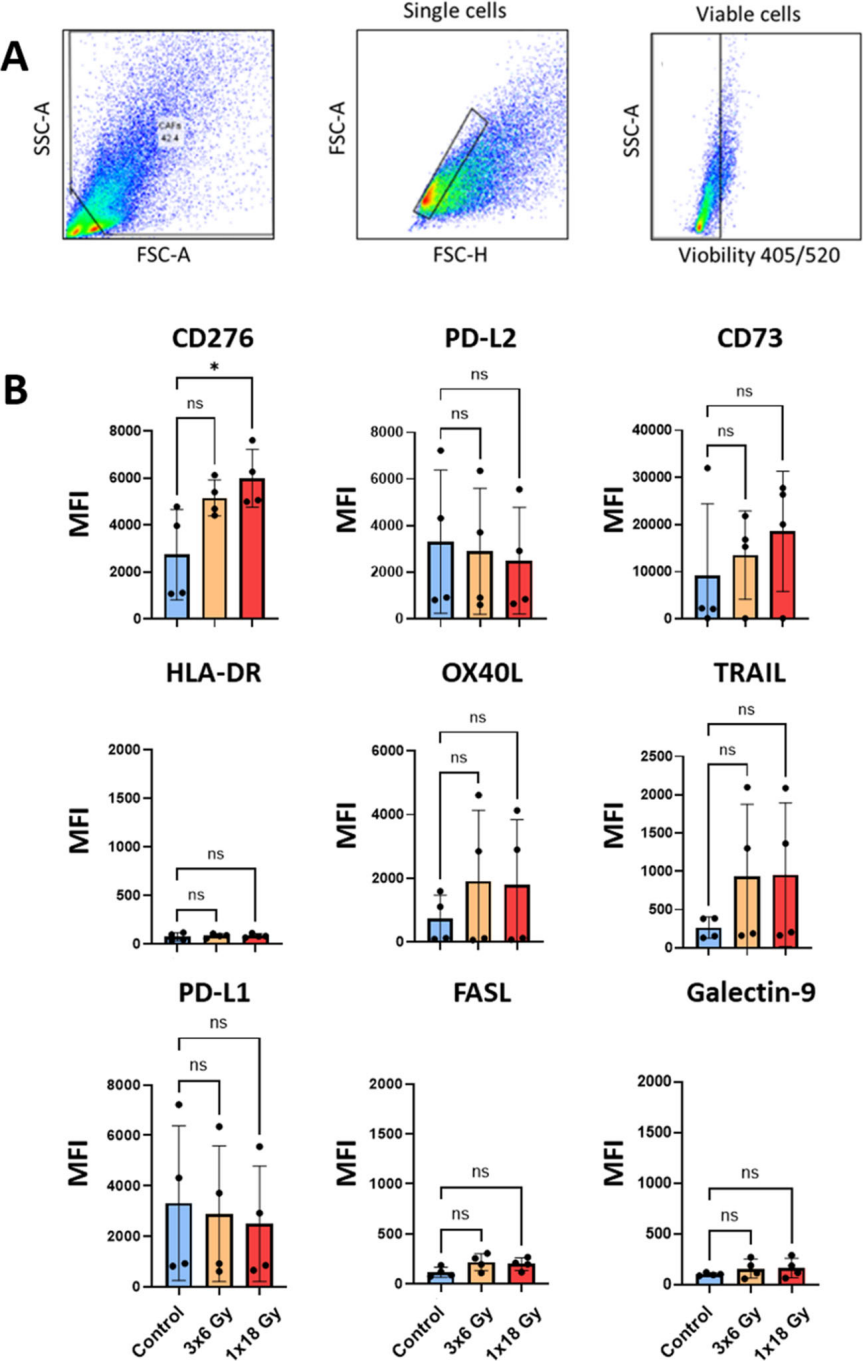
Surface expression of immunoregulatory receptors on irradiated Human lung CAFs. **(A)** Gating strategy used to analyze expression of immunoregulatory receptors on CAFs, 5 days after radiation treatment. **(B)** Bar graphs represent mean (± SD) values from flow cytometry analysis of 5 randomly selected CAF donors, measured independently. Results are expressed as median fluorescence intensity (MFI). Welch ANOVA test and *p-*values were determined between control and irradiated CAFs individually. *: Statistically significant, with p<0.05. ns: Not significant.

Besides the effects exerted by soluble signals, CAFs have the potential to regulate the function of effector immune cells via cell-cell mediated interactions. In this respect, we have analyzed radiation-induced changes in the cell surface expression of a number of immune checkpoint receptors/ligands and other receptors involved in immune regulation by flow cytometry, 5 days after radiation treatment, following the gating strategy described in **Figure 5A**. The results unveil that some relevant molecules in the context of immune therapy such as programmed death-ligand 1 (PD-L1) and PD-L2, and the receptor directly involved in immunosuppression CD178 (Fas ligand) are not significantly modified in radiation-induced senescent CAFs (**Figure 5**). Likewise, other relevant cell surface molecules, such as the antigen-presenting molecule HLA-DR, or galectin-9 (ligand for the checkpoint receptor TIM-3) also remain unaffected after cell irradiation (**Figure 5B**).

Interestingly, the surface expression of the ectonucleotidase CD73, which participates in the conversion of ATP into immunosuppressive adenosine is up-regulated in irradiated CAFs in a donor-dependent manner, both after (1x18Gy) and (3x6Gy) (**Figure 5**). Similarly, immunosuppressive receptors CD253 (TRAIL receptor), CD252 (OX40 ligand), and expression of the immune checkpoint molecule CD276 (B7-H7), which participates in T cell immunosuppression are also up-regulated in irradiated CAFs (**Figure 5**).

### Changes in the expression of CD73 and CD276 after radiotherapy in *in vivo* models

**Figure 6:**
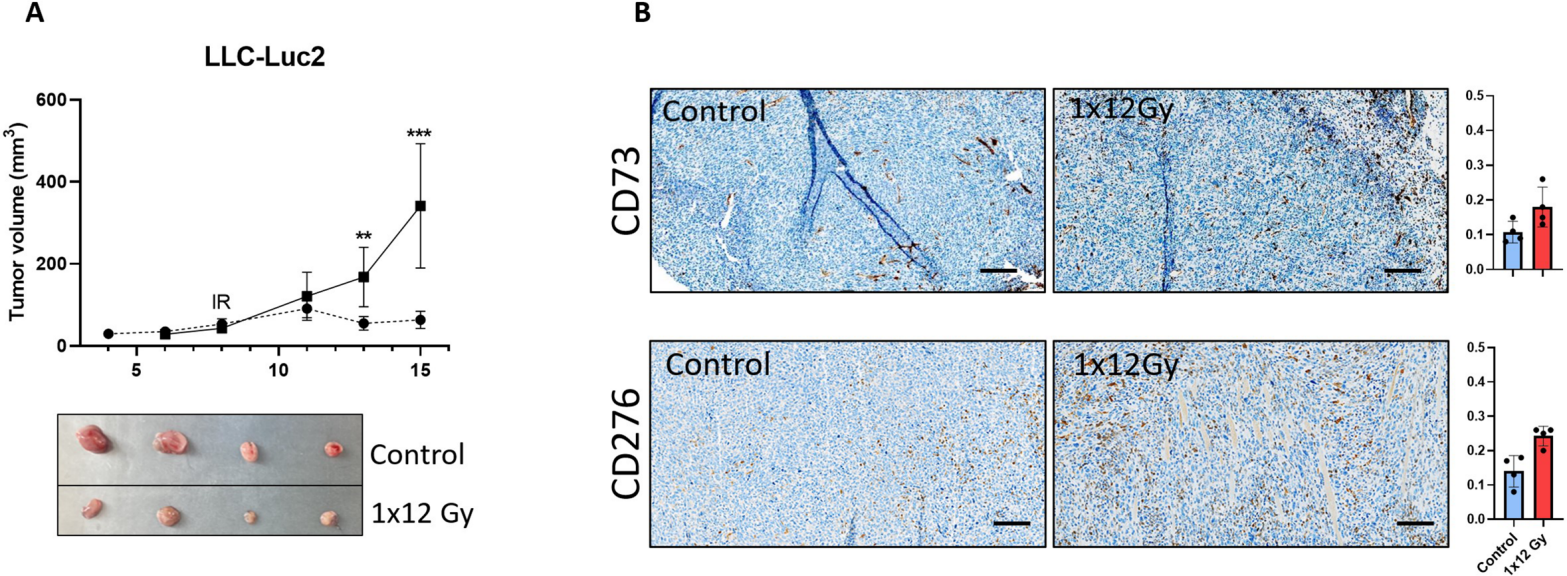
Radiation decreased tumor volume and induced expression of immunoregulatory receptors *in vivo*. Subcutaneous **(A)** LLC tumors from (1x12Gy) irradiated and non-irradiated (0Gy) Black6 mice (n=4 per group) were quantified as a percentage of total tumor area. **(B)** Immunohistochemistry was performed to assess the expression of CD73 and CD276 in stromal cells from these tumors. Results represent the mean (± SD) values from 4 different tumors per group. IR, irradiation. Graphs represent optical densities of 3,3′-Diaminobenzidine (DAB) staining of 10 different fields from 4 different mice per group. **:Statistically significant, with p<0.01. ***: Statistically significant, with p<0.001.

The observed changes in the expression of relevant immunosuppressive receptors in CAFs exposed to radiation *in vitro* (CD276 and CD73), prompted us to explore if such effects occurred also in *in vivo* tumor models. Mice irradiated with a single dose of 12 Gy showed a significant reduction in tumor volume compared to the non-irradiated group (**Figure 6A**). Results from immunohistochemistry (**Figure 6B**) demonstrate that the intra-tumoral expression of both CD73 and CD276 is enhanced in irradiated tumors one-week post-treatment, thus confirming that the observed effects of radiation are reproducible in *in vivo* situations.

## Discussion

Accumulating evidence during the last decade suggests that the anti-tumor effects of radiotherapy go beyond the direct damage exerted on DNA matter in malignant cells (45–47). Added effects of RT include release of reactive oxygen species (ROS) and other cytotoxic molecules from targeted cells that may kill also neighboring cells and the ignition of a tumor-specific immune response that exerts anticancer effects at the systemic level (abscopal responses) (38, 48). In the context of RT-induced anti-tumor immunogenicity, signals derived from cancer cells may contribute to both antigenicity and adjuvanticity, via the release of tumor-specific antigens and ICD signals respectively (38, 49). However, the contribution of irradiated non-malignant cells of the TME to anti-tumor adjuvanticity has been poorly documented. In this study, we present novel data on the contribution of irradiated CAFs, one of the most prominent elements of the TME, to the expression of immunogenic signals in irradiated tumors. Our data reveal, by various means, that CAFs do not undergo ICD-like cell decease, and do not contribute to the release of danger signals or interferon type-I responses following irradiation. Interestingly, the secretion of soluble immune regulatory cytokines and growth factors remain comparable between untreated and radiation-induced senescent CAFs. Of note, whereas expression of some immune checkpoint ligands remains unchanged, the cell surface expression of key immunoregulatory receptors such as CD73 and CD276 is upregulated in irradiated CAFs both *in vitro* and *in vivo*, which could influence therapeutic outcomes.

CAFs are known to be highly radioresistant cells in the TME, being able to survive ablative doses of radiation when established in *in vitro* cultures (24, 50). Our data confirm previous results on the radioresistant nature of CAFs and demonstrate that CAFs avoid apoptosis but become senescent after exposure to both single intermediate and high radiation doses. Some reported radiation effects on CAFs include activation of DNA damage responses and enhanced surface expression of integrins (24). In this study, we demonstrate also that both medium-high and single-high ablative radiation doses are able to induce micronuclei formation in CAFs. Intriguingly, the highest radiation dose (18Gy) was less efficient in triggering micronuclei formation on CAFs than a single dose of 6Gy. Micronuclei formation occurs primarily during mitosis at the time of cell division. The number of irradiated cells that are able to enter into cell cycle is higher at lower radiation doses and therefore, the occurrence of micronuclei may be paradoxically higher at intermediate radiation doses than at ablative radiation doses. Induction of cytosolic DNA and RNA in irradiated tumor cells is a crucial step for the initiation of IFN-I responses and the further activation of adaptive anti-tumor immunity (51). Despite the observed formation of DNA fragments (micronuclei) in some of the irradiated CAFs, we were unable to demonstrate IFN-I secretion in 20x concentrated CAF-conditioned medium collected at different time-points post-treatment. Likewise, levels of CXCL10 released into the culture medium by CAFs were rather low and remained largely unchanged after radiation exposure. Nevertheless, we observed enhanced expression of CXCL10 in CM from an individual CAF donor after exposure to 6Gy radiation doses, which points to patient-specific responses at intermediate radiation doses.

Among the described mechanisms involved in CAF-mediated immunomodulation, most effects are attributed to the release of soluble immunoregulatory/inflammatory mediators, including factors such as IL-6, IL-8, CXCL12/SDF-1, VEGF or TGF-β (21, 52). Additionally, in the context of radiotherapy, unrepaired DNA damage may lead to stabilization of p53 and activation of cytostatic programs that ultimately lead to cell senescence (53). Senescent cells in the TME may exhibit a senescence-associated secretory phenotype (SASP), which can contribute to increased local inflammation and potentially to promotion of cancer cell growth and survival (54–56). In this context, it is important to consider that release of mitochondrial DNA into the cytosol occurring in senescent cells has been found to activate the cGAS-STING pathway and to ultimately regulate SASP (57). In our study, we confirm that both intermediate and high radiation doses are able to induce cellular senescence in CAFs, which become permanently established some days post-treatment. Based on the established paradigms, we have compared the secretion of eleven different immunomodulators in the conditioned medium of untreated and radiation-induced senescent CAFs. Interestingly, we have not observed substantial differences in the release of any of the studied immunomodulators. This observation confirms similar data obtained in previous studies (29, 31, 58), and suggests that radiation exposure and senescence transformation is not able to overcome the already activated status of CAFs prior to treatment.

One of the well-described cytoprotective pathways driven by RT depends on the nuclear translocation of active NF-κB dimers (40). NF-kB is sensitive to a variety of intracellular environments enforced by RT, including DNA damage (via DDR kinases), cytosolic cGAS-STING activation, and oxidative stress (41, 59, 60). Moreover, the RT-driven secretion of inflammatory cytokines such as TNF-α and IL-1β may lead to activation of NF-kB signaling in target cells. On the other hand, the transcriptional programs coordinated by NF-kB exhibit considerable heterogeneity, depending on both the activation pathway and the cell type (40). In our study, we demonstrate that the NF-kB and the STAT pathways are not activated in CAFs upon radiation exposure, indicating that in this particular cell type, the NF-kB pathway may not represent a relevant cytoprotective mechanism, and may also explain the essentially unaltered secretion of immunomodulators observed from irradiated CAFs.

An alternative mechanism for regulation of tumor immunity is via direct cell-cell contact signaling, including cell surface receptors that are known to interfere with survival and/or activation status of effector immune cells (61). Among the referred cell surface molecules, we could highlight immune checkpoint ligands such as PD-L1, PD-L2, and galectin 9, cell death ligands such as CD253 (TRAIL) and CD178 (FAS ligand), or stimulatory molecules including CD80, CD252 (OX40L) or MHC molecules. Radiation has the potential to exert effects on surface expression levels of immune checkpoint receptors/ligands on tumor cells and other cells of the TME (62). Along the same line, irradiated cancer cells upregulate expression of a variety of NK cells-activating ligands (NKALs) on their surface as a consequence of ROS generation and activation of DDR kinases (63). In CAFs from NSCLC tumors, a recent study has demonstrated radiation-induced enhanced expression of receptors on CAFs which exert regulatory functions over NK cells (CD155 and HLA-E) after irradiation (30). In this study, we found that the expression of relevant immune checkpoint ligands such as PD-L1, PD-L2, Galectin-9, and other immune regulatory receptors such as Fas ligand remain unchanged after radiation exposure. On the contrary, expression of immune regulatory receptor TRAIL, OX40 ligand, expression of ectonucleotidase CD73, and immune checkpoint receptor B7-H3 (CD276) is enhanced on IR-induced senescent CAFs the ectonucleotidase CD73 and immune checkpoint receptor B7-H3 (CD276) is enhanced on IR-induced senescent CAFs. The enhancing effects of radiation on CD73 expression were particularly strong in some specific CAF donors. However, large variations in baseline expression of CD73 among donors were reducing the overall statistical significance of these results. The *in vitro* observations on CD276 and CD73 were confirmed in *in vivo* tumor models. This observation suggests that CAFs in the stroma of irradiated tumors may contribute towards the establishment of a more immunosuppressive microenvironment. A limitation of our study is the reliance on *in vitro* systems, where CAFs are cultured in monoculture. This experimental design restricts our ability to extrapolate CAF responses to more physiological *in vivo* conditions, where interactions with other cellular components in the tumor microenvironment might play a significant role. To address radiation-induced changes in CAFs within a tumor context, we attempted to isolate CAFs from previously irradiated tumors. However, isolation of CAFs from subcutaneously transplanted LLC tumors in mice has proven challenging, impeding our capacity to replicate the experiments conducted with primary cell cultures. Nonetheless, it is important to bear in mind that results from our study correspond to *in vitro* monocultures and that some of the observed outcomes may differ from RT-induced effects in the whole tumor context.

## Conclusions

**Figure 7:**
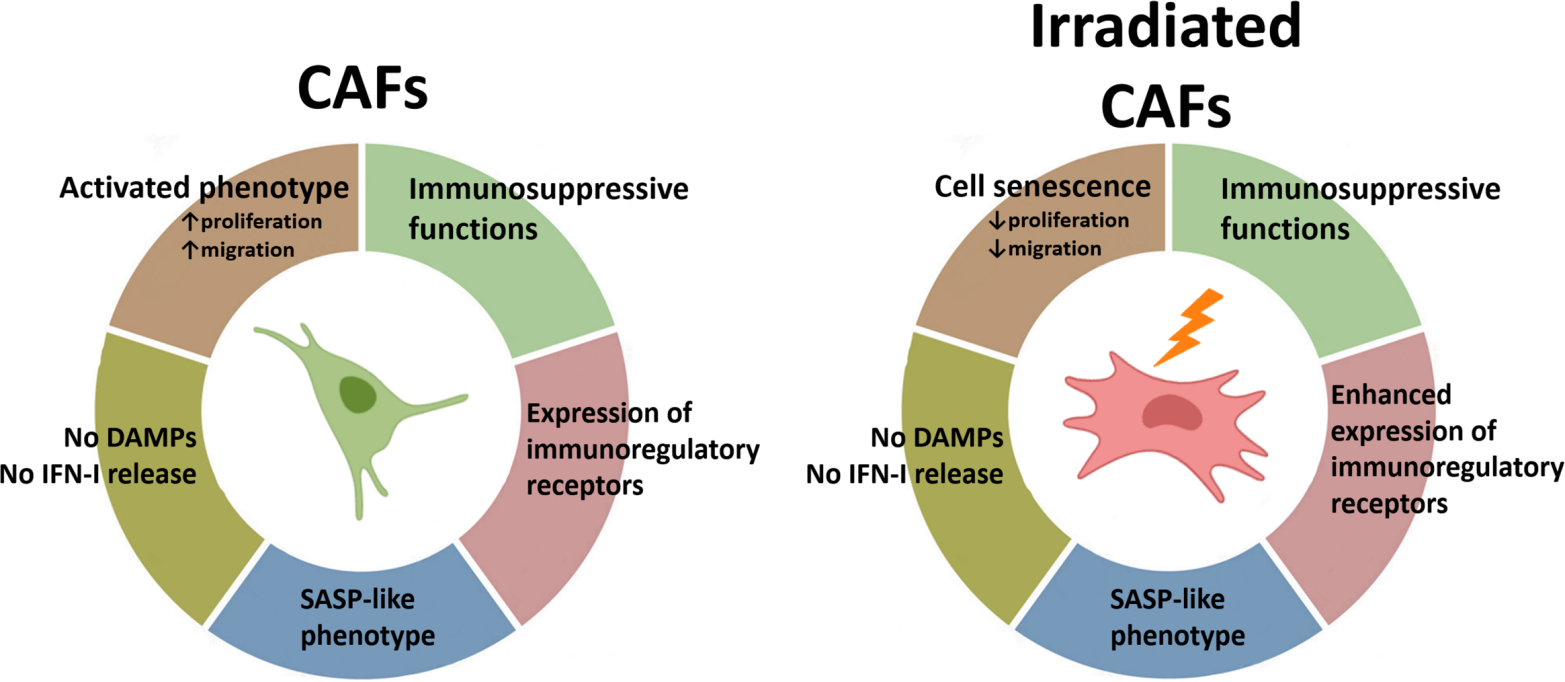
Schematic overview of findings: Radiation-mediated effects on immunological signals in CAFs. Radiation promotes the acquisition of a senescence phenotype in CAFs with a concurrent reduction in cell proliferation and migration rates. Exposure of CAFs to ablative radiation doses is insufficient to induce release of DAMPs. Likewise, radiation is unable to trigger measurable IFN-I responses in CAFs. CAFs are engaged in the release of soluble inflammatory and immunoregulatory factors resembling the senescence-associated secretory phenotype (SASP), and this phenotype is mostly unchanged after radiation exposure. Surface expression of some important immunoregulatory receptors in CAFs becomes increased after radiation exposure.

In the TME, not all the cellular components display the same radiosensitivity. While some cells such as tumor-associated macrophages (TAMs) or CAFs exhibit remarkable radioresistance, other cell entities such as endothelial cells and natural killer cells are more radiosensitive (38). Furthermore, the response of cells to RT-induced cellular stress is also affected by multiple factors including their proliferative state, the oxygen levels, their anatomical location, and their degree of differentiation (11). Observations from this study demonstrate that CAFs do not succumb to fractionated intermediate or single high radiation regimens but instead enter into a state of cell senescence. Despite the observed formation of intracellular DNA fragments (micronuclei) following radiation, CAFs do not undergo ICD-like cell demise and the cellular damage is insufficient to ignite IFN type-I responses. Of note, the acquisition of a stress-induced senescent phenotype is not followed by substantial changes in the SASP. However, the enhanced expression of some cell surface immunoregulatory molecules after radiation may contribute to unwanted elevation of immunosuppressive signals in the TME followed by RT (**Figure 7**). Hence, these results should be taken into consideration, especially in patients affected by stroma-rich tumors. Combinatory treatment strategies aiming to counteract adverse immunosuppressive signals, such as agents targeting the adenosine/CD73 system, could be considered to gain the full potential of RT as immunoadjuvant treatment.

## Data Availability

The original contributions presented in the study are included in the article/**Supplementary Material**. Further inquiries can be directed to the corresponding author.

## Supplementary material

Supplementary Figure 1 Uncropped scans of Western blots found in **Figure 2**.

Supplementary Figure 2 Uncropped scans of Western blots found in **Figure 3**.

Supplementary Figure 3 Uncropped scans of Western blots found in **Figure 4**.
