# Supplementary figures and images for: Immunological signatures from irradiated cancer-associated fibroblasts
Source: Front Immunol. 2024 Sep 6;15:1433237. doi: 10.3389/fimmu.2024.1433237 (PMC11412886; doi:10.3389/fimmu.2024.1433237)

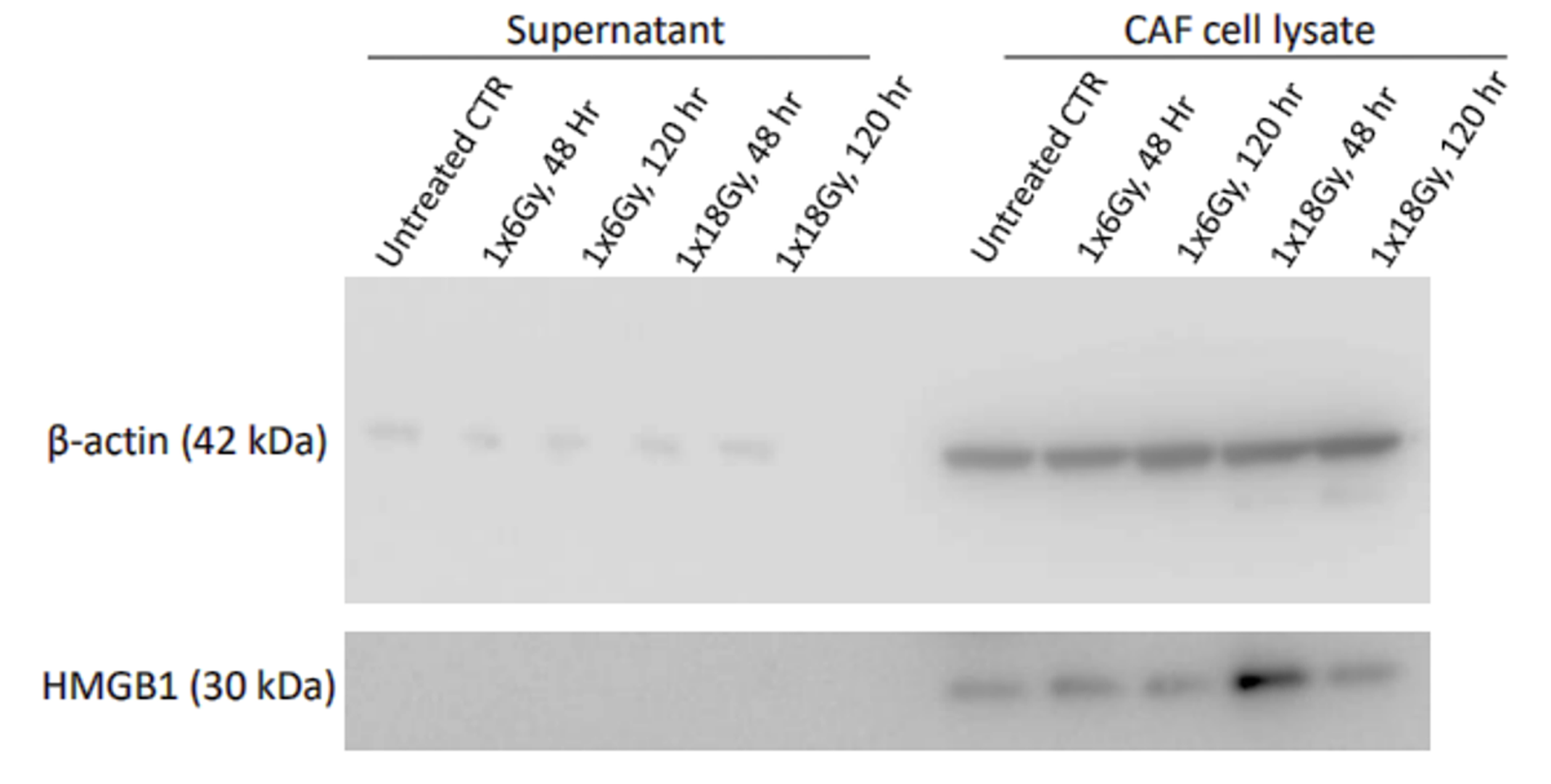

Supplement: Supplementary Figure 1 — Uncropped scans of Western blots found in Figure 2 . [file Image1.tif]

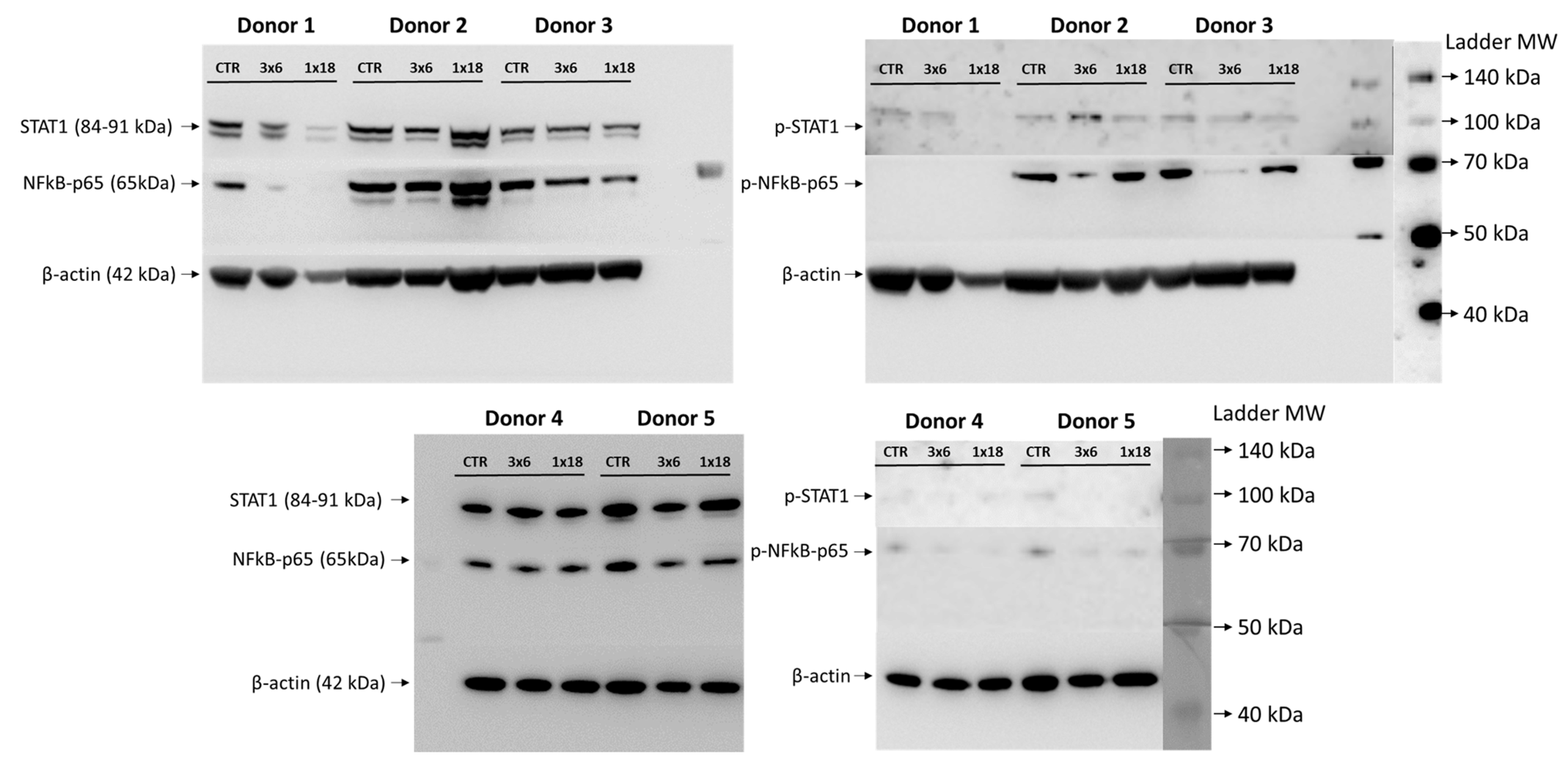

Supplement: Supplementary Figure 2 — Uncropped scans of Western blots found in Figure 3 . [file Image2.tif]

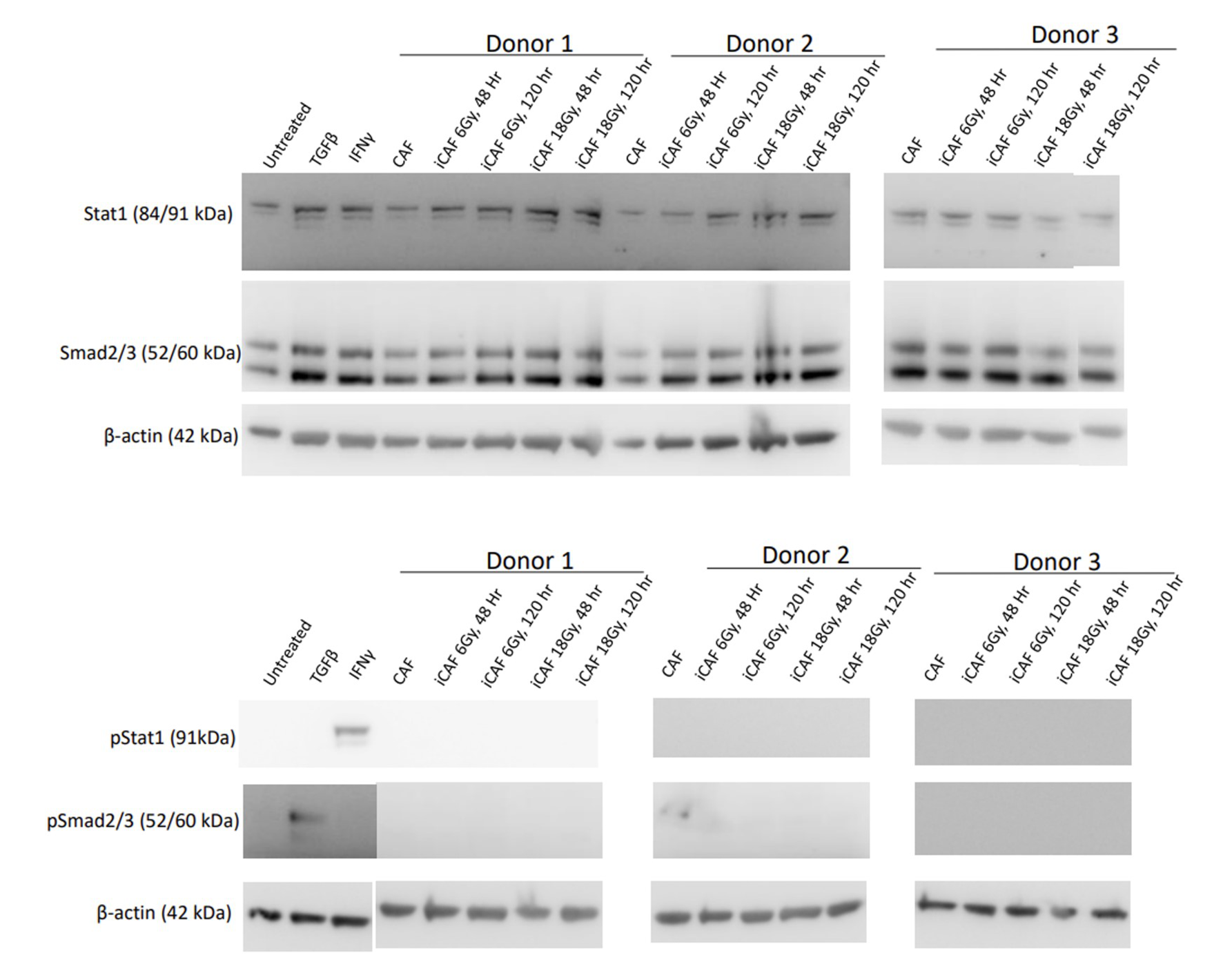

Supplement: Supplementary Figure 3 — Uncropped scans of Western blots found in Figure 4 . [file Image3.tif]
