# Supplementary material for: Immunological signatures from irradiated cancer-associated fibroblasts
Source: Front Immunol. 2024 Sep 6;15:1433237. doi: 10.3389/fimmu.2024.1433237 (PMC11412886; doi:10.3389/fimmu.2024.1433237)
Supplement: Supplementary file 4 [file Table1.docx]

**Supplementary Table 1:** Clinical and patient records corresponding to CAF donors and Donor-derived tumor cells used in this study.

| **Donor** | **Sex** | **Tumor type** | **T-size (mm)** | **Stage** |
| --- | --- | --- | --- | --- |
| CAF#1 | Female | Adenosquamous carcinoma | 60 | pT3N0Mx |
| CAF#2 | Male | Non-keratinized  squamous cell carcinoma | 24 | pT2N2Mx |
| CAF#3 | Male | Pleomorphic adenocarcinoma | 46 | pT2bN0Mx |
| CAF#4 | Male | Squamous cell carcinoma | 24 | pT1cN0Mx |
| CAF#5 | Female | Keratinized  squamous cell carcinoma | 42 | pT2bN0Mx |
