# Supplementary material for: Immunological signatures from irradiated cancer-associated fibroblasts
Source: Front Immunol. 2024 Sep 6;15:1433237. doi: 10.3389/fimmu.2024.1433237 (PMC11412886; doi:10.3389/fimmu.2024.1433237)
Supplement: Supplementary file 5 [file Table2.docx]

**Supplementary Table 2** - List of antibodies used for western blot and flow cytometry analysis. Antibodies used in this study are listed along with catalog numbers and concentrations used—WB=Western blotting; FC=Flow cytometry.

| **Protein** | **Antibody** | **Concentration** |
| --- | --- | --- |
| HMGB-1 |  | 1:1000 (WB) |
| Stat1 | Cell Signaling, #14994 | 1:1000 (WB) |
| Calreticulin | Abcam, # ab2907 | 1:100 (FC) |
| p-Stat1 | Cell Signaling, #9167 | 1:1000 (WB) |
| NFkB-p65 | Cell Signaling, #8242 | 1:1000 (WB) |
| p-NFkB-p65 | Cell Signaling, #3033 | 1:1000 (WB) |
| CD276 | Miltenyi, Cat # 130-118-704 | 1:50 (FC) |
| CD273 | Miltenyi, Cat # 130-116-685 | 1:50 (FC) |
| CD73 | Miltenyi, Cat # 130-112-061 | 1:50 (FC) |
| HLA-DR | Miltenyi, Cat # 130-111-941 | 1:50 (FC) |
| PD-L1 | Miltenyi, Cat # 130-122-815 | 1:50 (FC) |
| OX40L | BD Biosciences, Cat # 563766 | 1:50 (FC) |
| CD178 | Miltenyi, Cat # 130-118-087 | 1:50 (FC) |
| CD253 | Miltenyi, Cat # 130-119-384 | 1:50 (FC) |
| Galectin-9 | Miltenyi, Cat # 130-124-272 | 1:50 (FC) |
